# Application of the Hot Wire Method to Measure the Thermal Conductivity Coefficient of a Gypsum Composite

**DOI:** 10.3390/ma15196564

**Published:** 2022-09-22

**Authors:** Krzysztof Powała, Andrzej Obraniak, Dariusz Heim, Andrzej Mrowiec

**Affiliations:** 1 Faculty of Process and Environmental Engineering, Lodz University of Technology, 90-924 Lodz, Poland; 2 Polytechnic Department, Akademia Kaliska, 62-800 Kalisz, Poland

**Keywords:** phase change material, gypsum, paraffin, compressive strength, thermal conductivity, hot wire method

## Abstract

Currently, there is much discussion about modern technologies and solutions in construction. There are new solutions that save electricity or heat, usually in buildings additionally equipped with intelligent management systems. High hopes are placed on building materials. Every investment begins with them. The basic building materials include materials such as cement, bricks, hollow bricks or plasterboard, and their modification and the use of admixtures ensure the greatest changes in the parameters of the building. This article focuses on the preparation and testing of gypsum mortar consisting of gypsum, phase change material and polymer. The idea was to replace the proven method of adding microencapsulated phase change material by direct binding. This article presents the study of thermal conductivity by the hot wire method. Using this method, tests of temperature changes during plaster hardening were also carried out. Compressive strength tests were also carried out on the 14th, 21st, 28th, 35th and 105th day from the date of making the samples. For each of these tests, three types of samples with different polymer content were used. After a series of tests, the best results were obtained by a series of samples with 0.1% polymer.

## 1. Introduction

In the face of the deteriorating situation of the energy market, many companies are looking for modern solutions aimed at reducing the losses of already generated energy as much as possible. In the present situation, many constructions, not only industrial, but mainly residential buildings, lose heat energy in large amounts [1] in an uncontrolled manner. Therefore, building material specialists have noticed that much depends on how such materials are modified. One of the simplest ways is through different admixtures [2]. Thanks to such measures, the insulation properties of the material are improved [3] and the mechanical properties are changed [4]. This is important from the point of view of materials such as concrete or gypsum, which are the basic building materials [5].

The basic material that is used in every construction is gypsum. Gypsum is a mineral binder, obtained in the process of partial dehydration of natural gypsum stone from an opencast mine. Gypsum has been used for several hundred years in various forms. Its decorative advantages were noticed as early as the nineteenth century. According to the history, it was recognized that these were the beginnings of the production of plasterboard. The production was relatively easy and the production costs were low [6]. Currently, gypsum plays an important role in interior finishing in the form of gypsum putties or the aforementioned plasterboards, as well as gypsum plasters used outside the building. It has been found that such materials can be easily modified. Substances such as paraffin, fatty acids or hydrated salts [7,8] are used. These substances belong to phase change materials.

PCM is readily used. There are three known methods that allow the use of this material in the building layer. One of them is the use of microencapsulation, which covers the PCM particle with a layer of polymer. The PCM particles introduced in this way directly into the concrete or gypsum layer prevent the negative effects of this combination [9,10,11,12]. Another method that allows for the connection of the phase change material is the use of a porous polymer, thanks to which a material with a stabilized shape is created. The last method is the direct application of PCM, e.g., paraffin, to plaster or concrete. It is the least used method due to the many problems it causes. The most serious of these is the problem of paraffin leakage when changing the phase from solid to liquid. This not only has a poor visual effect, but it is also dangerous in terms of fire protection. In some cases, PCM is absorbed in porous materials as a result of capillary and surface forces. Another disadvantage resulting from the direct application of PCM is corrosion [13]. This mainly applies to reinforced concrete and PCM in the form of fatty acids.

However, despite several disadvantages, the phase change material has many useful advantages confirming the sense of its use in building materials. The main advantage is the storage of thermal energy during the phase change. This mainly applies to paraffin. In addition, it is worth noting that when changing the phase from solid to liquid, the heat capacity increases, which contributes to the storage of more thermal energy. The whole process ends with a change in the liquid state into solid, during which the heat energy that was previously stored is released. The entire process is synonymous with the day and night cycle. During the day, when the layer with PCM is exposed to temperature and sunlight, it stores energy as a result of the phase change of the material. When the evening hours approach, the material changes its phase again, returning energy to the room, and some of the heat energy produced by the heating system can be saved [14]. Additionally, the process can be easily predictable. This mainly concerns paraffins, as this material is produced with different melting points. This way, the process can be predicted and the PCM can be adjusted to the prevailing conditions to make it as effective as possible [15].

As mentioned before, microencapsulation is the best way to introduce PCM into a building material. There are many works in the literature on the use of PCM in the form of microencapsulation [16,17,18,19]. The method of incorporating PCM in this way completely eliminates the problem of leakage to the surface of the building layer. The microencapsulation process is carried out in many ways. These are physical, physico-chemical, and chemical methods. Depending on the advancement of the technology used, granules with a diameter of no more than 1 mm are obtained. Due to the polymer layer, PCM will not degrade during the phase change from solid to liquid [20]. However, due to the involvement of modern technologies in the production of granules, the microencapsulation process is extremely expensive, which may be unprofitable when used in buildings with large surfaces. Therefore, many scientists are looking for an alternative to this process. For this reason, this article is devoted to presenting such a solution.

The aim of this article is to present a composite consisting of gypsum, paraffin and a polymer that is a specific alternative to microencapsulation and other methods of combining gypsum with PCM. The study is a continuation of work on the problem detailed in [21] in which the appropriate proportions and materials were previously selected. This article focuses on determining how the addition of paraffin and polymer influenced the thermal and mechanical parameters. Initially, strength tests were carried out on the basis of three polymer shares: 0.1%, 0.5% and 1%. A hot wire method was used in the study, thanks to which temperature changes during gypsum setting were examined on the same samples with the same proportions. Finally, the hot wire method was used to determine the thermal conductivity coefficient. The study was made more attractive by changing the temperature by placing the samples in a climate chamber. Measurements were made at 10 °C, 13 °C, 16 °C, 18 °C, 20 °C, 22 °C, 24 °C, 26 °C, 28 °C, 30 °C, 32 °C and 35 °C. On the basis of the research, conclusions were drawn defining the suitability of a given composite for further research.

## 2. Materials and Methods

In this article, Rubitherm paraffin with the designation RT22HC was used. This marking also determines the point of transformation of the solid phase into the liquid phase at a temperature of 22 °C. The choice was clear, because as it turned out, the transformation temperature is close to the temperature of thermal comfort in the room. The composite is intended to be used in the production of plasterboard. Technical data are given in Table 1.

The gypsum used in the article comes from the Atlas company, which is a product of the Dolina Nidy mine. Raw gypsum was used in the research, without any admixtures shortening the drying time or increasing other parameters. Table 2 presents the most important properties of gypsum.

### 2.1. Preparation of the Composite in the Form of a Homogeneous Mixture

The first part of the study was to prepare a homogeneous mixture that is simple and does not generate high production costs. This method was supposed to be an alternative to expensive microencapsulation, which allowed the production of gypsum mortar for, e.g., plasterboard. However, in order for the gypsum composite to meet the conditions of use on a real scale, it was important to eliminate the phenomenon of paraffin leakage during the phase change. For this purpose, a polymer was used which was added in three percentages by weight, 0.1%, 0.5% and 1%, as in the previous study [21]. The procedure according to which the samples were made is shown below.

1. Water was added to the raw gypsum in a weight ratio of 0.35:0.65.
2. Liquid paraffins were added to the prepared gypsum in a weight ratio of 0.8:0.2.
3. The polymer was added to the prepared composite in three weight percentages: 0.1%; 0.5%; 1%.
4. The mixture prepared in this way was mixed on a magnetic stirrer for about 15 s until obtaining the appropriate consistency.

### 2.2. Compressive Strength Test

The compressive strength tests were the next stage. For this purpose, the Instron device (Instron, Norwood, MA, USA) was used, with which five series of measurements were carried out. Samples of 0.1%, 0.5% and 1% by weight of polymer were prepared. Each series consisted of 12 samples, making it possible to determine the average measurement. The compressive strength test was not carried out according to the applicable standards. This was due to the comparison of the results of the compressive strength measurement from the previous study [22], where the tests according to the PN-EN 13279-2: 2014-02 standard were carried out. In addition, 105-day long-term studies were performed in this work from the production of the samples. The study was carried out using the proprietary method, where 12 samples were used for one series for each type of sample. In addition, the dimensions of the samples were changed, differing from the dimensions described in the standard. This was because the accuracy of the results was improved by calculating the mean of multiple measurements. The samples were 2 cm × 2 cm × 2 cm. In each case of production, the samples, after taking them out of the molds, were placed in a closed room at a temperature of 21 °C in order to evaporate the moisture. Each series, after an appropriate hardening time, was sent for strength tests. Measurements were made 14, 21, 28, 35 and 105 days from the production of the samples.

### 2.3. Testing the Temperature Increase during Solidification by the Hot Wire Method

**Figure 1:**
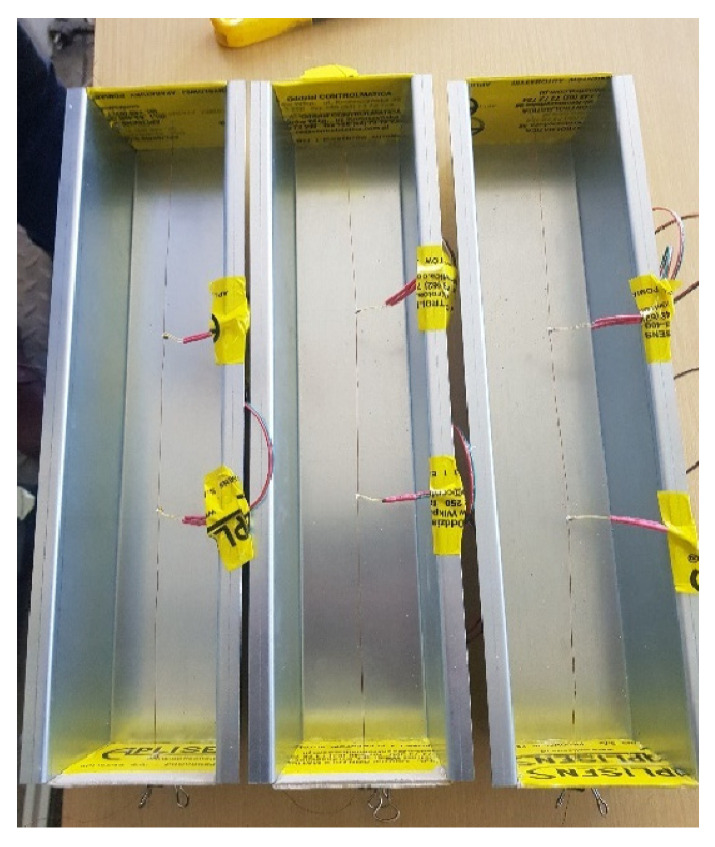
Gypsum molds with embedded wire.

In order to eliminate an undesirable change in temperature during setting, we checked how it changes depending on the proportion of polymer. We used the hot wire technique. This method is characterized by fairly high accuracy and the ability to measure various materials in different states of aggregation. There is no convection during the measurement, so the study can be carried out on most materials without distorting the result. The measurement was therefore carried out under the conditions of heating the material by determining the heating rate. This method was refined by the authors together with the entire apparatus and measurement accuracy [23]. For the needs of the research, appropriate plaster casting molds were prepared. The molds were 4 cm × 4 cm × 30 cm. Inside, there was a wire to which two Pt 500 temperature sensors were connected (Figure 1). Three samples were made with the weight fractions of 0.1%, 0.5% and 1%. Measurements were performed every 15 s.

### 2.4. Testing the Thermal Conductivity by the Hot Wire Method

The temperature changes during solidification were tested with the hot wire method. The study of the thermal conductivity coefficient using the same method is more complicated. As in the case of testing temperature changes during solidification, also in this case, 3 types of samples were used: with 0.1% polymer, 0.5% polymer and 1% polymer, with the difference being that the samples were allowed to dry for 2 weeks to remove moisture. Each sample, additionally during the measurement, was placed in a climatic chamber in which they were subjected to temperature changes. Thus, it was possible to carry out measurements for the temperatures 10 °C, 13 °C, 16 °C, 18 °C, 20 °C, 22 °C, 24 °C, 26 °C, 28 °C, 30 °C, 32 °C and 35 °C. Due to the fact that the hot wire method, used to test temperature changes during hardening and to measure thermal conductivity, is an innovative method, it was not assigned the standards according to which the tests were carried out.

This method was not used to measure the material containing the phase change material. Thanks to temperature conditioning, it was possible to check how the thermal conductivity coefficient changes. The measurement is therefore carried out in the conditions of heating the material indirectly by determining the heating rate. In the *T-lnt* system after the so-called transition period, the relationship becomes linear. The thermal conductivity is calculated by:

(1) $$\lambda =\frac{Q}{4\cdot \pi \cdot L}\cdot \frac{\ln\frac{t2}{t1}}{T2-T1}$$

where:

*Q*—the amount of heat emitted by the heat source (W);
*L*—heating element length (m);
*t*—time (s); and
*T*—temperature of the heating element (°C).

The amount of heat Q emitted by the heating element is equal to the amount of heat absorbed by the tested material in the unit time, assuming no loss and no accumulation heat in the heating element.

(2) Q=U·I

where:

*U*—voltage (A); and
*I*—current intensity (V).

To carry out the measurements, an analog temperature transducer was used, which was connected to a computer measuring station consisting of an input multifunctional 14-bit A/C converter type NI USB-6009 from National Instruments (Austin, TX, USA) and interactive software for recording and measurements. The LAbView program (LabVIEW Professional Development System, NI, Austin, TX, USA) was used to query the results.

## 3. Results

### 3.1. Compressive Strength Test of a Homogeneous Gypsum Composite

**Figure 2:**
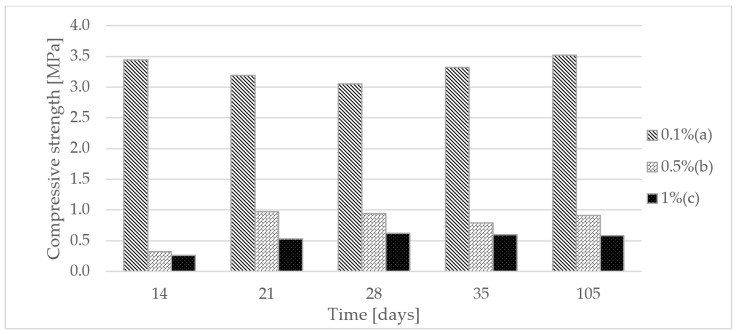
Compressive strength of gypsum samples depending on the polymer content (a) 0.1%; (b) 0.5%; (c) 1%.

In order to determine the usefulness of the presented composite, it was necessary to examine the strength tests. Therefore, the samples were subjected to compressive strength tests. The test was carried out in room conditions with a temperature of 22 °C and medium humidity. In Figure 2, it can be seen that the samples with 0.1% polymer in each test series had the highest compressive strength. As expected, the sample achieved its highest strength on day 105 from its manufacture. It was similar with the 0.5% and 1% polymer samples, with the difference being that the 0.5% polymer sample on day 35 slightly decreased in strength, which resulted from the large number of samples where the average value was calculated (Table 3).

### 3.2. Testing the Temperature Increase during Solidification by the Hot Wire Method

**Figure 3:**
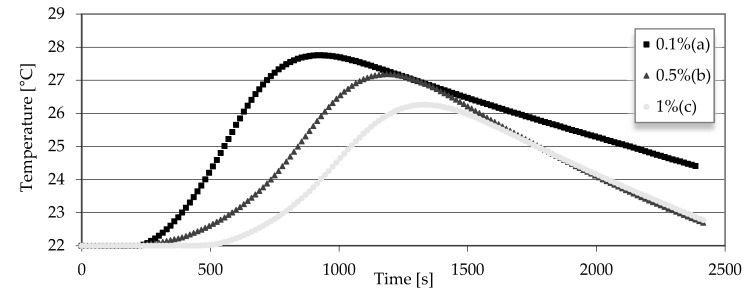
Dependence of reaching the maximum temperature and hardening time of gypsum samples with polymer (a) 0.1%, (b) 0.5% and (c) 1%.

The hot wire method for measuring temperature changes as the composite dries up has proven effective. The test began from the moment of pouring gypsum on the molds until the initial temperature was reached. Figure 3 shows that the proportion of the polymer contributes to the temperature increase as the composite solidifies. It can be seen that not only the peak temperature value, but also the time is shifted when the highest temperature is reached. It can be seen that the 1% polymer sample reaches a temperature 1.5 °C lower than the 0.1% polymer sample. In addition, the time is almost 1000 s behind the highest temperatures between these samples being reached.

Analyzing the results in Figure 3, the trends in the delay in reaching the highest temperature and the time in which this temperature are reached is clearly visible. A correlation can be noticed between this test and the measurements from the Vicat apparatus from the previous article [24]. Table 4 shows the correlation, i.e., that samples with a higher polymer content solidify longer than samples with a smaller amount of polymer. For comparison, a sample with 0.1% polymer has a drying time about 3 times faster than a sample with 1% polymer.

### 3.3. Testing the Thermal Conductivity by the Hot Wire Method

**Figure 4:**
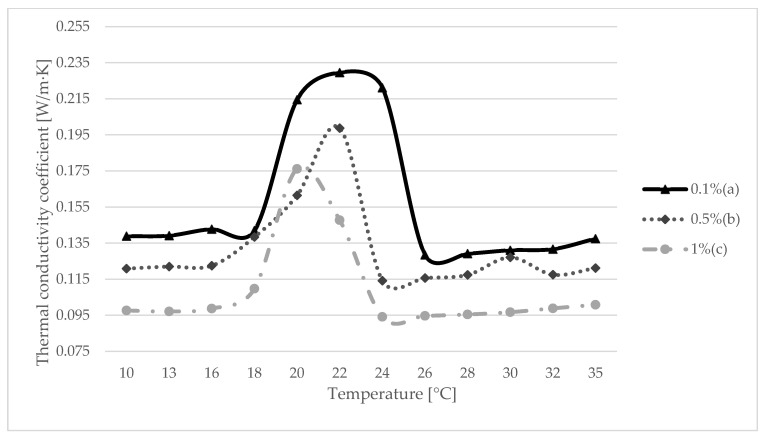
Coefficient of thermal conductivity depending on the proportion of polymer (a) 0.1%, (b) 0.5% and (c) 1%.

The test was carried out for three samples with the content of 0.1% polymer, 0.5% polymer and 1% polymer. Due to the large number of measurements, each sample took two weeks test. In Figure 4, the results of the conducted thermal conductivity test can be seen. The best results were obtained with a sample with 0.1% polymer, reaching 0.23 W/m·K exactly at the point of phase transition of the paraffin used (22 °C). The sample with the content of 1% of polymer turned out to be the worst, reaching slightly more than 0.17 W/m·K. It is also worth noting the achievement of the maximum value of the thermal conductivity coefficient, because each of the samples reached 22 °C at the point of the phase transition. The only deviation was noted for the 1% polymer sample, where the maximum was at 20 °C (Table 5).

## 4. Discussion

There are many intelligent building materials that significantly influence the performance of a given building. In the discussed studies, a gypsum composite was described, which is to increase the temperature parameters of a given layer. The main purpose of a given mixture is to introduce it into a plasterboard, thanks to which it is possible, to some extent, in a given temperature range, to achieve favorable heat storage results. However, for this to happen, the parameters of the building must be determined, and then the parameters of the rooms that will allow the proper operation of such a board. In this study, a thermal conductivity test, which is extremely important from the point of view of energy storage speed, allowed us to determine whether a given material absorbs energy well from the environment. On the basis of the research, a series of results were obtained (Figure 4), which allowed us to determine in which temperature range the composite behaves best. By analyzing Figure 4, it can be concluded that the given phase-changing material (paraffin) correctly adjusted to the changes in the measurement interval. Not without significance was the fact that the paraffin with the transformation temperature of 22 °C was selected. Thanks to the study, it was possible to determine the thermal conductivity coefficient, which turned out to be the best for the sample with 0.1% polymer and amounted to 0.23 W/m·K. We must take into account the sense of choosing a given PCM. Due to the use of plasterboard inside the room, the given temperature was chosen because it is close to the temperature of thermal comfort. This means that PCM will change its aggregate state from concentrated to liquid, thus storing thermal energy. This is important given the time of the highest activity of the PCM due to the temperature range. Too low a transformation temperature will cause a rapid phase transformation, and thus the maximum of the stored energy will be quickly reached. Moreover, the temperature of the layer in which the PCM has been applied may not decrease to the extent that the phase change occurs again, and then the heat dissipation inside the building will be small. Then, the use of PCM no longer makes sense. The situation will also be similar when the transformation temperature is too high, and consequently, there will be little chance for a phase change, and thus for the correct amount of stored energy.

Another important aspect in the design of a given layer using PCM is the percentage. Depending on the amount of PCM, subsequent efficiency during the process is important. As in the case of choosing the right temperature, the right amount of PCM is important, which has an impact not only on the process itself, but also on its suitability in construction. The aesthetic value is not without significance in this case, but also the achievement of the required compressive strength. Some sources say [25] that the best share is 30% and gives the best thermal effects. A larger amount reduces these parameters and there are significant problems with endurance. Based on previous research on the composite [21], it was found that in a given solution described in this article, 20% PCM gives the best results. However, this solution had consequences that needed to be addressed. First of all, there was some leakage of paraffin during the transformation. This is unacceptable; therefore, the polymer that gave the best results in terms of paraffin retention was used. In this article, strength tests were carried out to check how the compressive strength changed. A series of samples with 0.1% polymer content turned out to be the best. Moreover, on each day of the measurement, this series exceeded 2 MPa, even though the composite layer may be considered as an insulating layer, which, according to the PN-EN 13279-1 standard, should not be subject to any strength requirements.

Another important factor that is subject to the PN-EN 13279-1 standard is the hardening time of the gypsum composite. It is obvious that each admixture of a different substance affects the hardening time, and the same was the case here. The average drying time of the plaster is 5–7 min depending on the conditions and the ratio of water to plaster. In this case, a 19 min drying time was achieved. Thanks to the possibility of studying temperature changes using the hot wire method, the drying time tests were compared to temperature changes. Comparing Figure 3 and Table 4, one can notice the dependence on the length of the drying time to the increased temperature. It can be seen that there was a heat evolving reaction for the 0.1% polymer sample, reaching almost 28 °C, and thus, the drying time was the shortest.

Comparing the relationships between individual studies, it can be seen that the sample with 0.1% polymer content, compared to the other samples, turned out to be the best. This proves that the results achieved in the previous articles and the choice of 20% paraffin content is the best choice. The results achieved in this article are a continuation of work on the composite and constitute part of a doctoral dissertation on this subject.

## 5. Conclusions

Based on the conducted research, it was possible to draw the following conclusions:

- The addition of the polymer influenced the compressive strength of the gypsum samples. Looking at the results, a sample with 0.1% polymer gave favorable strength results. In the first studies, between days 14 and 28, endurance decreased, while at day 105, it reached the highest value. This means that the gypsum is mature enough to meet all the regulations that allow the use of gypsum composite in construction.
- Measurement of temperature changes as a function of time using the hot wire method showed that the proportion of polymer reduces the maximum temperature and lengthens the time to reach it. Moreover, it also coincides with the results from the Vicat apparatus, which were presented in another article.
- A hot wire heat conductivity study confirmed that the material used gave the best results when the phase transition point was reached. This means that when thermal comfort is achieved in the room, the composite that will be used to cover the wall layer will be activated and will store energy.

When analyzing the results of the conducted research, it can be noticed that the best results are achieved by a sample with 0.1% polymer. It is possible that thanks to such a small amount of polymer, there was a significant improvement in strength, and thus a significant improvement in thermal parameters. This means that the pores have been filled to such a level with a small amount of polymer that subsequent amounts do not give comparable results. However, in order to determine whether this actually happened, microscopic photos are necessary. Despite these uncertainties, the direct addition of paraffin to plaster is extremely simple and can be an alternative to other methods. The presentation of the hot wire method for the study of thermal conductivity is also noteworthy for gypsum. Despite the level of complexity and numerous calculations, it gives reliable results. There is reason to believe that this method, after refining and reducing the measurement time, gives high hopes for using other mixtures and composites for measurements.

## Figures and Tables

**Table 1:** Main parameters of Rubitherm RT22HC paraffin.

Parameter	Unit	Result
Melting area Congealing area	°C °C	20–23 23–20
Heat storage capacity	kJ/kg	190
Specific heat capacity Density solid Density liquid	kJ/kg·K kg/L kg/L	2 0.76 0.7

**Table 2:** Main parameters of Atlas gypsum.

Parameter	Unit	Result
Calcium sulfate hemihydrate content	%	>95
(β-CaSO4·0.5H_2_O)		
Crystallization water	%	5.6–6.0
Mechanical strength after drying to constant weight		
- for bending		>5.0
- for compression	MPa	>12.0

**Table 3:** The results obtained during the compressive strength test.

	0.1% (a)		0.5% (b)		1% (c)	
Day	Average	Standard Deviation	Average	Standard Deviation	Average	Standard Deviation
-	(MPa)		(MPa)		(MPa)	
14	3.44	0.57	0.32	0.12	0.26	0.05
21	3.19	0.52	0.97	0.53	0.53	0.14
28	3.05	0.58	0.94	0.65	0.62	0.10
35	3.32	0.82	0.79	0.42	0.60	0.12
105	3.52	0.70	0.91	0.39	0.58	0.12

**Table 4:** Results of drying time of individual samples.

Percentage of Polymer	Drying Time
0.1%	19 min
0.5%	38 min 42 s
1%	55 min 30 s

**Table 5:** Results of the thermal conductivity coefficient for individual samples of gypsum composite depending on the polymer content.

	0.1% (a)	0.5% (b)	1% (c)
Temperature	Average		
(ºC)	(W/m·K)		
10	0.138872	0.120914	0.09772
13	0.139181	0.122027	0.097125
16	0.142789	0.122518	0.09873
18	0.141993	0.138395	0.109752
20	0.214557	0.161498	0.176168
22	0.229555	0.198744	0.147769
24	0.22113	0.114198	0.09413
26	0.128469	0.115705	0.094686
28	0.129188	0.117409	0.09549
30	0.131072	0.127101	0.096732
32	0.131698	0.117528	0.098869
35	0.137517	0.121184	0.100865

## Data Availability

Not applicable.
